# Impacts of tuberculosis services strengthening and the COVID-19 pandemic on case detection and treatment outcomes in Mimika District, Papua, Indonesia: 2014–2021

**DOI:** 10.1371/journal.pgph.0001114

**Published:** 2022-09-30

**Authors:** Trisasi Lestari, Kamaludin, Christopher Lowbridge, Enny Kenangalem, Jeanne Rini Poespoprodjo, Stephen M. Graham, Anna P. Ralph

**Affiliations:** 1 Center for Tropical Medicine, Universitas Gadjah Mada, Yogyakarta, Indonesia; 2 Global and Tropical Health, Menzies School of Health Research, Charles Darwin University, Darwin, Northern Territory, Australia; 3 Timika Research Facility, Papuan Health and Community Development Foundation, Timika, Indonesia; 4 Mimika District Health Office, Timika, Indonesia; 5 Mimika District Hospital, Timika, Indonesia; 6 University of Melbourne Department of Paediatrics and Murdoch Children’s Research Institute, Royal Children’s Hospital, Melbourne, Australia; 7 The Burnet Institute, Melbourne, Australia; 8 Royal Darwin Hospital, Darwin, Northern Territory, Australia; Universidad Nacional de Colombia, COLOMBIA

## Abstract

Indonesia is a high-burden tuberculosis (TB) country with a wide case detection gap, exacerbated by the COVID-19 pandemic. We aimed to review the epidemiology of TB in a high-endemic setting of Indonesia before and during the implementation of health system strengthening activities for TB, including during the first two years of the COVID-19 pandemic. We analysed TB program data from Mimika District, Papua, Indonesia from 2014 to 2021. Health system strengthening activities to improve the programmatic management of TB were implemented from 2017 onwards. Activities included decentralization of TB services, training and mentoring of healthcare workers, improved screening for co-morbidities, and introduction and optimisation of Xpert testing in 2018. A total of 11,803 TB cases were notified to the Mimika District Health Office over eight years (2014–21). Between 2015 and 2019, there was a 67% increase in annual case notifications, an 89% increase in bacteriologically confirmed cases and the proportion of TB cases detected in primary care increased from 26% to 46%. In 2020, coinciding with the COVID-19 pandemic, investigation of people with presumptive TB fell by 38%, but the proportion of those tested with Xpert increased. TB case notifications decreased by 19% from 1,796 in 2019 to 1,461 in 2020, but then increased by 17% to 1,716 in 2021. Routine screening for co-morbidities (HIV, diabetes) among TB patients improved over time and was not affected by the pandemic. Treatment success overall was 71% and remained relatively unchanged. Loss to follow-up and death were 18% and 3.7% respectively. Improvements in TB case finding were observed over a period in which a range of health system strengthening activities were implemented. While COVID-19 had a negative impact on the TB program in Mimika District, there are encouraging signs of recovery. Further work is needed to improve TB treatment outcomes.

## Introduction

Tuberculosis (TB) is a leading infectious cause of death, although rivalled in recent years by SARS-CoV-2 (COVID-19). Indonesia has the second highest TB burden globally with an estimated 845,000 TB cases and 98,000 deaths each year [1]. There have been improvements in TB case detection in recent decades, especially since a major case detection gap was identified by the National TB Prevalence Survey in 2014–2015. In Indonesia, overall TB case notifications rose by 69% from 331,703 in 2015 to 562,049 in 2019 but a wide case detection gap remains due to a combination of underdiagnosis and underreporting of people diagnosed with TB. Indonesia accounted for 10% of the estimated 2.9 million TB cases not detected or notified globally in 2019 [1]. In 2021, only 47% of the estimated TB cases in Indonesia were notified [2]. Within Indonesia, there are diverse determinants of TB epidemiology, and variation in performance of regional TB programs. Mimika district in Indonesia’s easternmost province of Papua is a multi-cultural mining district with an annual TB case notification rate of 818 per 100,000 population in 2019 [2]. Details of TB epidemiology and program performance in Mimika have been scant, although past reports have indicated a very high burden of disease [3, 4].

In 2017, in a collaboration between the Mimika District TB Program, University of Gadjah Mada, Timika Research Facility and Menzies School of Health Research (Australia), we commenced a TB health-systems strengthening project, the Stronger Health Systems for multidrug-resistant tuberculosis and malaria (STRATUM) initiative and a follow-up initiative called PRIME-TB (Papua New Guinea and Indonesia for the Micro Elimination of TB). The focus of this project was on prevention of TB through implementation and scale-up of screening household contacts of people with TB and use of TB preventive treatment (TPT) among young (<5 years) child contacts without TB. However, a broader set of health system strengthening activities were also implemented, including improvements to TB program information management systems, and use of a Continuous Quality Improvement (CQI) framework to identify gaps and opportunities for improving the clinical and public health management of TB [5] Mimika district has also received TB program support through United States Agency for International Development (USAID) funded initiatives, including promotion of active case finding and technical assistance to develop a district action plan for TB control [6].

Since early 2020, the COVID-19 pandemic has impacted the delivery of TB services. Staff and resources were diverted to the pandemic response and TB patients have avoided health care contact to limit their exposure to COVID-19. Utilization of primary and tertiary health care decreased dramatically [7] Indonesian national TB case notifications dropped to 393,323 in 2020, a 31% reduction compared to 2019 and less than half the estimated caseload [1]. In response to escalating COVID-19 cases in Mimika from March 2020 the local government implemented large-scale social restrictions [8]. Unintended impacts on access of the population to healthcare, including TB care, quickly become evident.

We aimed to evaluate the impact of strengthening of TB services on TB case notifications, diagnostic practices and treatment outcomes in this high-incidence setting of Indonesia, as well as the impact of the first two years of the COVID-19 pandemic on any progress made.

## Methods

### Study design, setting and population

We conducted a retrospective epidemiological review of routinely collected TB program data. The study setting was Mimika District, located in Papua Province of Indonesia. The population of Mimika was 311,969 in 2020. It is a fast-growing district with urban and rural areas built around a copper and gold mine. It is a culturally and linguistically diverse area, with approximately half the population comprised of Indigenous Papuan peoples. There are five hospitals and 23 primary health centers, 20 of which provide TB diagnosis and treatment services, 13 located in rural settings [9]. Presumptive TB cases are requested to provide two sputum samples for Xpert assay (if available) or for smear microscopy if Xpert not accessible. Chest x-ray is only available in hospital and not routinely done for presumptive TB cases from primary health centers. The study population was limited to persons diagnosed with TB and notified to the TB program of Mimika District, Papua Province, Indonesia between 2014 and 2021.

### Data sources and variables

The National TB Program (NTP) of Indonesia uses manual paper-based data recording using national standardized forms to report all presumptive and confirmed TB cases. These data have been entered into an electronic platform that was first introduced into Mimika district in 2013, namely the ‘Integrated Tuberculosis Information System’ or Sistem Informasi Tuberkulosis Terpadu [SITT] for 2014 until 2020. It was then upgraded to the ‘Tuberculosis Information System’ or Sistem Informasi Tuberkulosis [SITB], which included additional details and forms, and option for real-time updates. Limited internet coverage in Mimika means data entry is often delayed and batched until TB staff can visit a site with internet access. The district TB coordinator regularly reviews submitted data and checks for completeness and consistency.

We used data from individual TB patient records and the electronic registers (SITT and SITB) for the period 1 January 2014 to 31 December 2021, and data on all people suspected of and investigated for TB for the period 1 January 2019 to 31 December 2021. Cohort data of treatment outcomes were documented up to 31 December 2020. To allow for delayed entry, data were extracted until March 2022. We undertook comprehensive data validation for TB patients by cross-checking paper forms with the electronic databases to minimize missing data, errors, and duplication. Ethnicity is not documented; therefore, we manually assigned ethnicity as indigenous Papuan or non-Papuan Indonesian on the basis of surname [10]. Surname is considered a reliable indicator of ethnicity locally. History of TB treatment was often misreported and was corrected by manual cross-checking. The main data source was the standardized individual TB register form (‘TB03’) which provides a record of patients who received TB treatment and their treatment outcome. Data collected included demographic characteristics, name of health facility, date of registration, date of treatment commencement, laboratory results, TB disease classification, HIV and diabetes status, treatment for drug-susceptible or drug-resistant TB, and treatment outcome. Extra-pulmonary TB (EPTB) is defined as TB disease involving organs other than the lungs. A case with evidence of TB in both pulmonary and extra-pulmonary sites is notified to TB program as pulmonary TB. Outcome was categorized as ‘treatment success’ if treatment cure or completion was recorded.

Only sputum specimens were evaluated for Xpert or smear, so no EPTB cases were bacteriologically confirmed. Laboratory results comprised smear microscopy for acid fast bacilli and nucleic acid amplification using the Xpert MTB/RIF (Cepheid, USA) (Xpert). Mycobacterial culture was largely unavailable. TB diagnosis in children is mostly a clinical diagnosis made using the ‘Indonesian pediatric TB scoring system’ (S4 Table); although limitations of this are recognized [11, 12]. Since 2016, Xpert has been recommended as the preferred first-line diagnostic option for samples from children with presumptive TB [11].

### Interventions to improve TB case finding

To understand local contextual factors impacting TB program performance in addition to our own health system strengthening work [5], we sought information from the Mimika District Health Office about TB activities led by government and non-government organisations during the years of the study. These are shown in Table 1. In summary, in 2016 the Community Empowerment of People Against Tuberculosis (CEPAT) project was introduced to improve TB case finding, monitoring and evaluation, and TB surveillance [6]. This project recruited and trained local community health workers to do door-to-door TB symptom screening and to facilitate attendance of symptomatic individuals at a Primary Health Centre (PHC, known as Puskesmas) for evaluation and laboratory testing. In 2017, the TB Challenge project provided technical assistance to the district TB coordinator and staff in health facilities to optimize TB reporting, public-private mix in TB management, and develop a district action plan for TB control [6]. The CEPAT and TB Challenge projects ended in 2018 and 2019, respectively.

**Table 1:** Health system activities introduced over time in Mimika District, 2014–2021.

Year	Activity
**2014**	1. Electronic data entry for TB (SITT) in use for a full year after installment in 2013 2. Routine TB monitoring and evaluation meetings were conducted biannually
**2015**	Treatment was initiated for the first drug-resistant TB case
**2016**	Introduction of TB CEPAT (‘Community Empowerment of People Against Tuberculosis’)–funded by USAID
**2017**	1. TB CEPAT project continued 2. TB Challenge project was introduced–funded by USAID 3. TDRRCI project (‘Tropical Disease Research Regional Collaboration initiative’)–funded by the Australian Government (the Indo-Pacific Centre for Health Security of the Department of Foreign Affairs and Trade) to support following activities: • Establishment of household contact screening and management with TPT using 6H for young (<5 years) child contacts under-5 years in five facilities • Local TB training, focusing on childhood TB and TPT • Introduction of quarterly Continuous Quality Improvement (CQI) meetings for TB program
**2018**	1. TB CEPAT project ended 2. GeneXpert MTB/RIF machine (first in the district) installed in the District Hospital 3. TDRRCI project activities: • TB training provided–TB treatment; child TB; TB in pregnancy • Scale-up of household contact screening and TPT program to 11 health facilities • TB program competition between health facilities with prizes for best-performing facilities–case finding, contact screening, HIV testing, TPT • Diabetes screening kit was distributed to health facilities • TB Monitoring and Evaluation meetings quarterly
**2019**	1. TB Challenge project ended 2. The electronic TB Data Entry was updated (SITB) 3. STRATUM project (‘Stronger Health Systems for multidrug-resistant tuberculosis and malaria’)–funded as a follow-up to TDRRCI by the Indo-Pacific Centre for Health Security of the Australian Government Department of Foreign Affairs and Trade to support: • Comprehensive care introduced for drug-resistant TB care • Scale-up of household contact screening and TPT program to 16 health facilities • Introduced monthly meetings for TB monitoring and evaluation, led by the District Health Office • TB training provided: infection control; treatment of infection
**2020**	1. Public health response to COVID–diversion of health services and human resources; isolation/curfew at home from 2 pm; reduced clinic time at facilities; the GeneXpert machine in the district hospital temporarily used for SARS-CoV-2 diagnosis; contact screening implemented for COVID instead of TB 2. A new GeneXpert machine installed in a Primary Health Center for TB diagnosis 3. STRATUM project and follow-up PRIME-TB project (’Papua New Guinea & Indonesia for the Micro Elimination of TB’), also funded by Australian Government (the Indo-Pacific Centre for Health Security of the Department of Foreign Affairs and Trade): • Introduced online TB monitoring and evaluation meeting • Introduced online TB training • Activities to strengthen detection and treatment of MDR TB
**2021**	1. Indonesian NTP introduced short regimen for TPT using 3HP and 3RH 2. Three additional GeneXpert machines installed (for a total of five in Mimika District): one to the district hospital for TB diagnosis; one to the district hospital for SARS-CoV-2 diagnosis during the national sport event; and one to a primary health center for TB diagnosis 3. PRIME-TB activities: • Hybrid, online and onsite, TB monitoring and evaluation meeting • Hybrid, online and onsite, TB training, consultation, and mentoring

TB: tuberculosis; SITT: Sistem Informasi Tuberkulosis Terpadu; SITB: Sistem Informasi Tuberkulosis; TPT: Tuberculosis preventive treatment; 6H: daily isoniazid for 6 months; 3HP: weekly isoniazid and rifapentine for 3 months; 3RH: daily rifampicin and isoniazid for 3 months; NTP: National Tuberculosis Program

From 2017, our research team implemented a multi-component intervention funded by the Australian government to initiate and strengthen household contact screening and management [5]. This project chiefly aimed to increase TB case finding among contacts and to provide TB preventive treatment (TPT) to young child contacts. Screening for co-morbidities was also supported with the distribution of blood glucose testing kits in 2018 to screen for diabetes and encouragement to offer HIV screening. Our project’s interventions have included group training, developing and providing educational materials, technical assistance and mentoring. Regular CQI meetings have been held to review progress and discuss achievements, barriers and challenges. All TB staff participate in the CQI meetings, raise their concerns and propose ideas to solve the identified problems.

A four-module GeneXpert (Cepheid, USA) machine was procured by the NTP for the district hospital in mid-2018 to detect *Mycobacterium tuberculosis* and rifampicin resistance (using ‘Xpert MTB/RIF’ cartridges) (Table 1). A second GeneXpert machine was installed in March 2020 at the Puskesmas with the highest TB caseload and three additional machines were installed in 2021, two at the district hospital and one at a PHC facility.

### The COVID-19 response in Mimika District and impact on TB activities

Two pandemic waves occurred during the study period. The first, in 2020, triggered strong social restriction policies [7]. The second, between May and September 2021 brought limited social restrictions [13]. By end of 2021, a total of 110,079 COVID-19 cases had been reported from Mimika district [14]. To support COVID-19 diagnosis, the Indonesian Ministry of Health recommended using Xpert Xpress SARS-CoV-2 cartridges [15, 16]. Therefore, the GeneXpert machine at the district hospital was temporarily diverted from TB to COVID-19 testing. Restrictions enforced during 2020 included reduced opening hours for healthcare facilities. COVID-19 stigma resulted in people avoiding contact with healthcare providers and rejection of outreach activities from TB staff. Most healthcare staff were diverted to the COVID-19 response, including the District TB Coordinator and TB staff in primary care and hospitals. In October 2021, Papua province hosted a national sports event with participants from around Indonesia. Healthcare workers, including TB staff in Mimika district, were required to do COVID-19 screening of participants and provide healthcare at the sports venues. This directly affected provision of routine care.

During the first pandemic year the STRATUM TB program maintained CQI meetings and TB training videoconferences and webinars. This allowed additional TB staff from rural areas with internet access to participate. Coordination and communication were also maintained using the WhatsApp group mobile messaging platform to keep TB staff informed and motivated. Since 2021, when social distancing was relaxed, ongoing training and CQI meetings were run using a hybrid in-person and online model.

### Analysis

Data were managed in Microsoft Excel 365 and analysis was performed using Stata v13 (StataCorp 2013. Stata Statistical Software: Release 13. College Station, TX: StataCorp LP). Among TB patients, we used Pearson’s Chi-square test to compare outcomes between subgroups with p<0.05 considered significant. Treatment outcomes were assessed by calculating odds ratios in univariable and multivariable models including age, ethnicity, residential location, health facility type, location of TB, mode of diagnosis, history of TB treatment, HIV and diabetes status. Longitudinal comparisons were tested with Stata’s *ptrend* command (chi-square statistic for trend) excluding the year 2014 due to incomplete data. To examine COVID-19 impact, we created an annotated plot of monthly numbers of potential (suspected) TB cases as well as TB case notifications from 2019 to 2021.

### Ethical approval

We obtained ethical clearance from the institutional review boards at the Universitas Gadjah Mada (KE/0715/06/2018; KE/1188/10/2019; and KE/0090/02/2021), the Northern Territory Department of Health and Menzies School of Health Research (2017–2777) and Charles Darwin University (H20110). Permission to access TB data was obtained from the Mimika District Health Office.

## Results

### Investigation of people with presumptive TB

**Fig 1:**
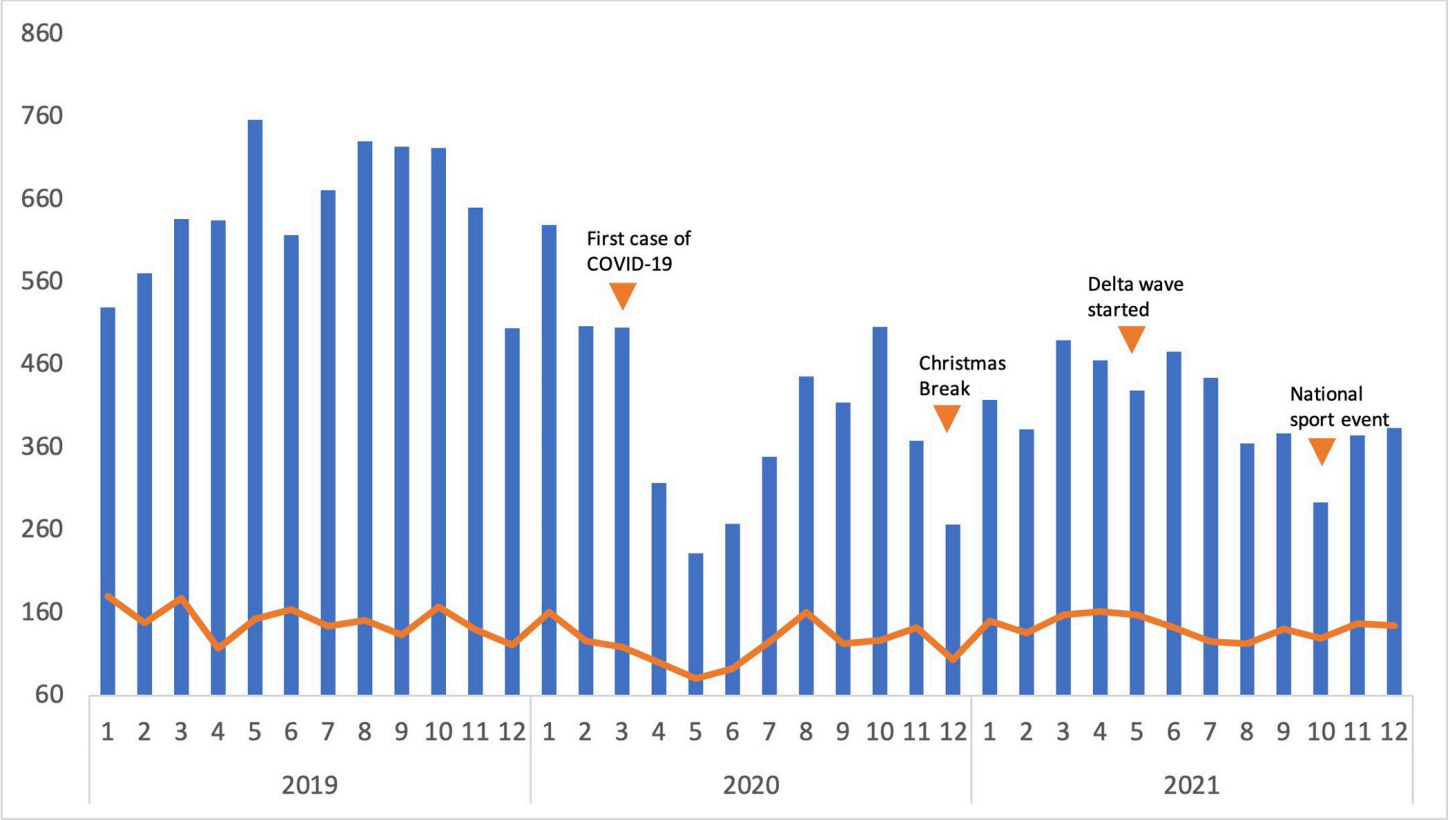
Monthly number of TB suspects and newly diagnosed patients with TB before and during the COVID-19 pandemic.

Data on screening for TB were available for 2019–2021. In 2019, 7745 people were screened for TB. This represented three quarters of the annual target of 10,314 set by the national TB program [17]. The impact of COVID-19 is shown as an annotated plot in Fig 1 and S1 Table; there was a 38% decrease in numbers of people screened for TB to 4808 in 2020, attributable to lockdown, drops in presentations to clinics and a fall in contact screening activities to identify potential cases. This coincided with an increase in the proportion of Xpert assays that tested positive, from 14.8% of community members with presumptive TB in 2019 to 19.5% in 2020 and 20.5% in 2021.

### TB notifications and characteristics of people with TB

During the eight-year study period, 11,803 TB cases were notified in Mimika District. Table 2 lists characteristics of TB cases by year. Overall, 69.1% were of Papuan ethnicity and 82.1% were from an urban setting, reflecting the general Mimika population [18]. The prevalence of co-morbidities among those tested were 10.5% with HIV infection and 5.4% with diabetes which compares to a prevalence of 2.4% and 1.1% in the general population respectively [18]. Overall, 22% of TB cases were EPTB, higher than the national average of 9% [1] and more common in children (27.3%), people living with HIV (26.2%) and Papuan people (24.7%).

**Table 2:** Characteristics and treatment outcomes of TB cases notified in Mimika District, Papua, Indonesia, 2014–2021.

Year of reporting	2014	2015	2016	2017	2018	2019	2020	2021	Total
Total cases	920	1078	1506	1567	1759	1796	1461	1716	11803
Characteristics	Number (%)								
**Sex**									
Male	520 (56.5)	616 (57.1)	865 (57.4)	913 (58.3)	1011 (57.4)	985 (54.8)	822 (56.3)	968 (56.4)	6699 (56.8)
Female	400 (43.5)	462 (42.9)	641 (42.6)	654 (41.7)	748 (42.6)	811 (45.2)	639 (43.7)	748 (43.6)	5104 (43.2)
**Age group**									
0–4 years	58	53	137	201	228	185	153	234	1249
	6.3	4.9	9.1	12.8	13.0	10.3	10.5	13.6	10.6
5–14 years	113	138	195	159	193	211	158	169	1336
	12.3	12.8	12.9	10.2	11.0	11.8	10.8	9.9	11.3
15–44 years	624	680	873	934	1027	1066	882	981	7067
	67.8	63.1	58.0	59.6	58.4	59.4	60.4	57.2	59.9
45–64 year (n)	111	183	264	240	280	298	239	308	1923
	12.1	17.0	17.5	15.3	15.9	16.6	16.4	18.0	16.3
> = 65 year (n)	14	24	37	33	31	36	29	24	228
	1.5	2.2	2.5	2.1	1.8	2.0	2.0	1.4	1.9
**Ethnicity**									
Papuan	632	799	1037	1043	1227	1266	1044	1151	8159
	68.7	74.1	68.9	66.6	69.8	70.5	68.7	67.1	69.1
Non-Papuan	274	250	391	450	521	525	451	565	3427
	29.8	23.2	25.9	28.7	29.6	29.2	30.9	32.9	29.0
Unknown	14	29	78	74	11	5	6	0	217
	1.5	2.7	5.2	4.7	0.6	0.3	0.4	0	1.9
**Previous TB**									
History of TB treatment	44	51	87	148	185	177	151	179	1022
	4.8	4.7	5.8	9.4	10.5	9.9	10.3	10.4	8.7
**Presenting facility**									
Primary care	303	277	468	584	762	822	739	772	4727
	32.9	25.7	31.1	37.3	43.3	45.8	50.6	45.0	40.1
Hospital	617	801	1038	983	997	974	722	944	7076
	67.1	74.3	68.9	62.7	56.7	54.2	49.4	55.0	59.9
**Residence**									
Urban Mimika	735	869	1213	1291	1467	1455	1199	NA	8249
	84.2	80.8	80.7	82.4	83.4	81.0	82.2		82.1
Rural Mimika	137	202	290	276	283	339	254	NA	1784
	15.7	18.8	19.3	17.6	16.1	18.9	17.4		17.7
Other district	1	4	0	0	9	2	5	NA	21
	0.1	0.4	0.0	0.0	0.5	0.1	0.3		0.2
**Site of TB**									
Pulmonary	723	792	1107	1226	1378	1232	1247	1526	9231
	78.6	73.5	73.5	78.2	78.3	68.6	85.3	88.9	78.2
Extra pulmonary	197	286	399	341	381	564	214	190	2572
	21.4	26.5	26.5	21.8	21.7	31.4	14.7	11.1	21.8
**HIV status**									
Negative	337	661	763	954	1290	1214	1052	1103	7374
	36.6	61.3	50.7	60.9	73.3	67.6	72.0	64.3	62.5
Positive	74	146	166	124	125	78	101	81	895
	8.0	13.5	11.0	7.9	7.1	4.3	6.9	4.7	7.6
Not known	502	252	535	469	333	436	255	482	3534
	55.3	25.1	38.3	31.2	19.6	28.1	21.1	31.0	29.9
**Diabetes mellitus**									
Negative	NA	NA	NA	485	1420	1242	1088	1356	5591
				30.9	80.7	69.2	74.5	79.0	67.4
Positive	NA	NA	NA	43	75	71	61	60	310
				2.7	4.3	4.0	4.2	3.5	3.7
Not tested	NA	NA	NA	1039	264	483	312	300	2398
				66.3	15.0	26.9	21.3	17.5	28.9
**Treatment outcome**									
Treatment success	475	766	1078	1183	1306	1280	1090	NA	7178
	51.6	71.1	71.6	75.5	74.3	71.3	74.6		71.2
Died	21	32	28	32	79	97	88	NA	377
	2.3	3.0	1.9	2.0	4.5	5.4	6.0		3.7
Loss to follow-up	182	210	261	285	308	344	229	NA	1819
	19.8	19.5	17.3	18.2	17.5	19.2	15.7		18.0
Failed	9	3	10	7	18	17	9	NA	73
	1.0	0.3	0.7	0.5	1.0	1.0	0.6		0.7
Transfer out	25	62	105	60	39	52	29	NA	372
	2.7	5.8	7.0	3.8	2.2	2.9	2.0		3.7
Not recorded	208	5	24	0	9	6	16	NA	268
	22.6	0.5	1.6	0.0	0.5	0.3	1.1		2.7

NA = not available

**Fig 2:**
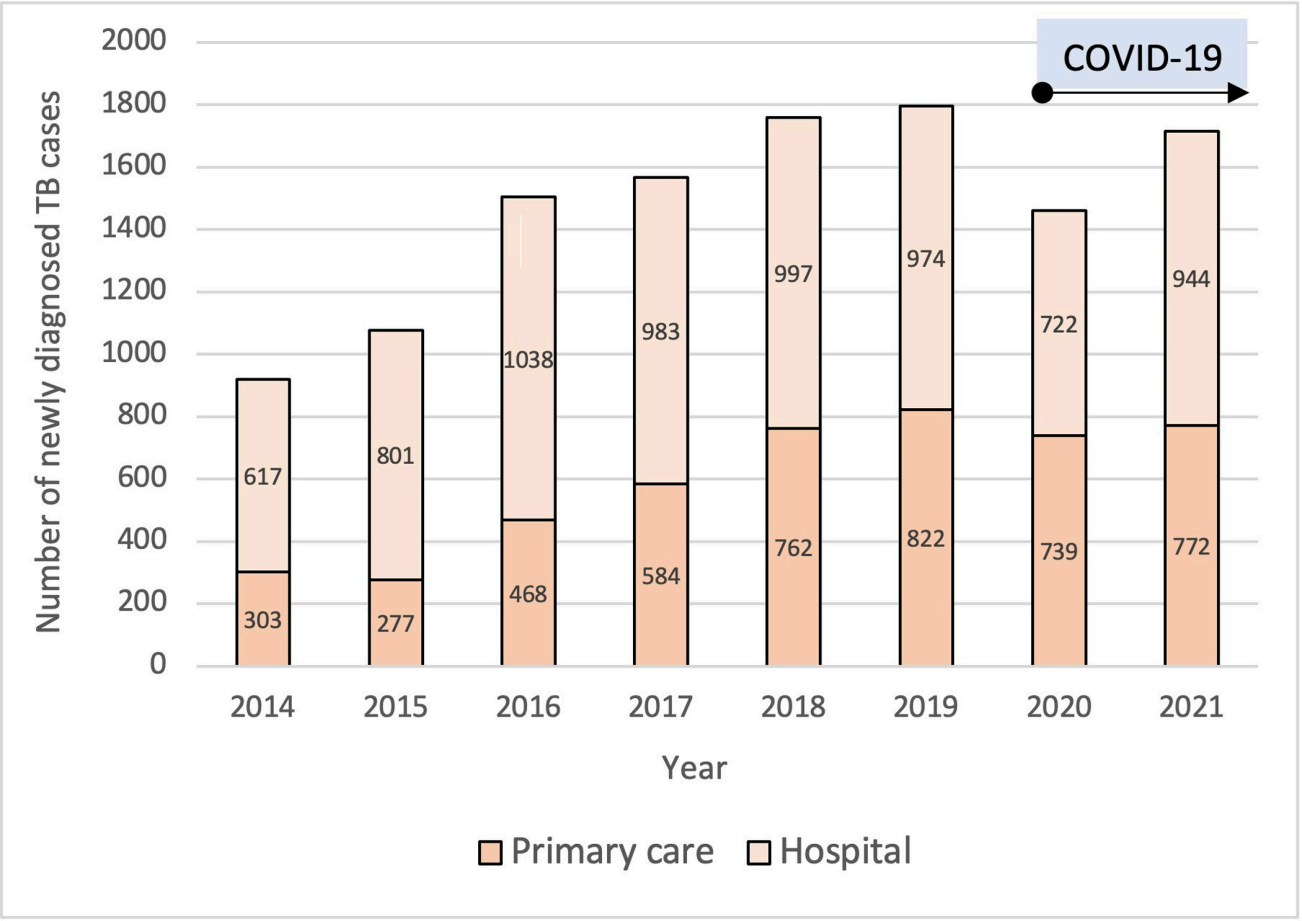
Number of newly diagnosed patients with tuberculosis in Mimika district from 2014 to 2021.

TB case detection and reporting increased annually until the onset of the COVID-19 pandemic in 2020 (Fig 2). Between 2015 and 2019 there was a 67% increase in notified TB cases (1078 to 1796 cases), achieving 94% of the estimated annual target case detection rate of 1910 cases for 2019 as set by the health authority. This was followed in 2020, coinciding with COVID-19 spread and lockdown measures, by a 18.7% decrease in case notification from 1796 in 2019 to 1461 in 2020, which then increased to 1716 in 2021.

### Diagnosis and quality of care among people diagnosed with TB

**Fig 3:**
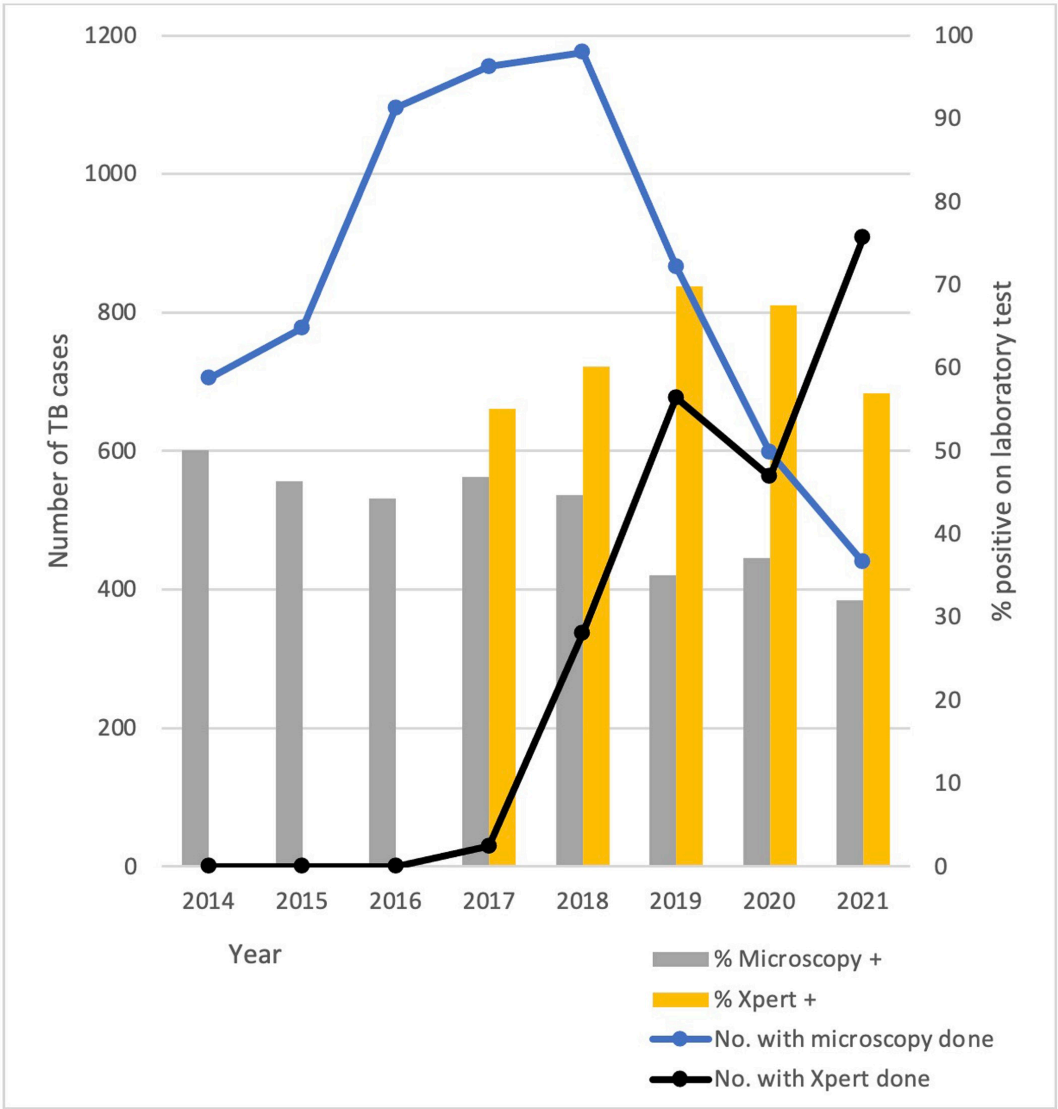
Smear microscopy and GeneXpert testing showing number of cases with testing performed (left y axis), and proportion of tests done which tested positive (right y axis).

The proportion of cases diagnosed in primary care increased over time from around a third to half of all cases (Fig 2). Although the proportion with bacteriological confirmation remained relatively constant at 35.8% overall, there was an 89% increase in the number of individuals with bacteriologically confirmed TB from 361 in 2015 to 682 in 2019 (Tables 3 and S1). All samples tested were sputum samples. The mode of diagnosis shifted from smear microscopy as the sole diagnostic option to Xpert being used in more than half the cases during the study period (Tables 3 and S1 and Fig 3). Bacteriological confirmation was uncommon in children (<15 years of age) but there was an increase each year in the proportion of children with laboratory confirmation from 1.5% in 2016 to 8.6% in 2020 (Table 3). This occurred as the proportion of children tested with Xpert increased; with diagnostic yield in children of 32.1% (62/193) positive on Xpert compared with 26.5% (56/211) for smear microscopy. Bacteriological confirmation in adults was 55.0% overall, again with Xpert having a higher diagnostic yield than smear microscopy.

**Table 3:** Bacteriological confirmation in people diagnosed with tuberculosis, 2014–2021.

Year	2014	2015	2016	2017	2018	2019	2020	2021	Total
**Number of TB patients**	920	1078	1506	1567	1759	1796	1461	1716	11803
**Bacteriologically confirmed N, %**	353	361	486	545	620	682	574	609	4230
	38.4	33.5	32.3	34.8	35.3	38.0	39.3	35.5	35.8
Diagnostic used* N, %									
Microscopy performed	705	778	1096	1155	1176	866	598	440	6814
	76.6	72.2	72.8	73.7	66.9	48.2	40.9	25.6	57.7
Microscopy positive	353	361	486	542	526	304	222	141	2935
	38.4	33.5	32.3	34.6	29.9	16.9	15.2	8.2	24.9
Xpert performed	NA	NA	NA	29	337	677	563	908	2519
				1.9	19.2	37.7	38.5	52.9	21.3
Xpert positive	NA	NA	NA	16	203	472	380	517	1587
				1.0	11.5	26.3	26.0	30.1	13.4
Rif-resistant	NA	NA	NA	6	13	36	21	26	102
				37.5	6.4	7.6	5.5	5.0	6.4
Xpert and Microscopy performed	NA	3	2	26	201	181	43	111	567
Xpert and Microscopy positive	NA	3	2	16	127	137	31	61	377
		100	100	61.5	63.2	75.7	72.1	54.9	66.5
**Age groups N, %**									
0–14 years	5	3	4	10	23	25	21	24	115
	1.4	0.8	0.8	1.8	3.7	3.7	3.7	3.9	2.7
> = 15 years	348	358	482	535	597	657	553	585	4115
	98.6	99.2	99.2	98.2	96.3	96.3	96.3	96.1	97.3

*All samples tested were sputum, including induced sputum in children.

Clinical diagnosis was made in 64.2% of total cases. Of 7,573 with a clinical diagnosis, 43.7% were adults with pulmonary TB, 23.7% were adults with EPTB and 32.6% were children. Of adults with clinically diagnosed pulmonary TB, only 4.7% had no microbiological testing undertaken; the rest (95.3% had a negative laboratory test (Xpert or smear). Among adults with presumptive pulmonary TB who seeks care at the hospital, 75.6% had chest x-ray. Clinically diagnosed TB cases were treated as per standard treatment regimens for drug-susceptible TB. The Indonesian pediatric scoring system was used to support clinical diagnosis in children without bacteriological confirmation with 53.3% recording a score and the majority (95.4%) of the scores recorded being 6 or more.

**Fig 4:**
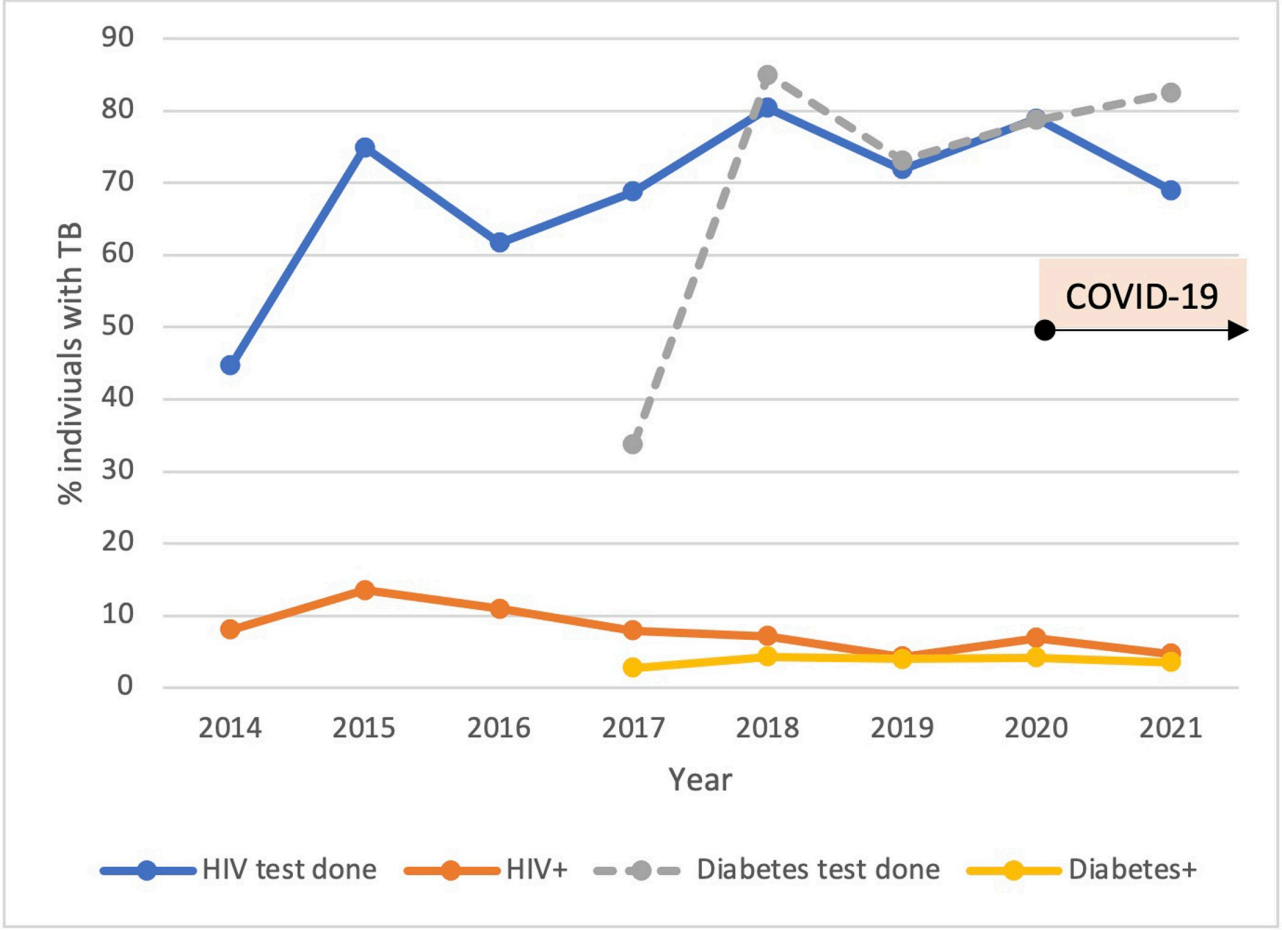
Proportion of people with TB in whom HIV and diabetes were tested.

Xpert identified 107 cases of rifampicin-resistant (likely multidrug-resistant) TB (S1 Table). Prior to the introduction of Xpert into District facilities, only samples from TB cases in which there was a high likelihood of multidrug-resistant TB on clinical suspicion were sent for Xpert testing at the distant reference laboratory situated in the provincial capital. Once Xpert testing became available locally and indications for Xpert testing broadened, the prevalence of rifampicin resistance among TB cases detected by Xpert was 5–7% between 2018 and 2021. The proportion of TB patients tested for HIV and diabetes improved during the study period and mostly remained strong during the pandemic years (Fig 4).

### Treatment outcomes

Documentation of treatment outcomes improved after 2014 and treatment outcomes up until the 2020 cohort are listed in Table 2. Treatment success did not significantly change over time, ranging from 71.1% to 74.6% between 2015 and 2020 (χ^2^ test for trend p = 0.21). A significant increase in the proportion of deaths was seen over time from 3% in 2015 to 6% in 2020 (p<0.001) and a fall in the proportion reported as lost to follow-up in recent years (Table 2). A rise in deaths of older individuals (>64 years) is noted in 2020 coinciding with COVID (S2 Table and S2 and S3 Figs). Treatment success rates were significantly higher in children (78.1%) compared with adults (69.2%), and in people treated in primary care (77.5%) compared with hospitals (67.1%) (p<0.001) (Table 4 and S1 Fig). One in five TB cases who initiated treatment in hospital were lost to follow-up, compared to one in ten whose treatment was initiated at a PHC (Table 2). People with TB/HIV co-infection had significantly lower treatment success (65.0%) than people without HIV (74.4%) (p<0.001) but ART status of those with TB/HIV was not routinely recorded. People with past TB had lower treatment success than those without (p = 0.001) (Table 4). Ethnicity was not associated with treatment outcome.

**Table 4:** Tuberculosis treatment outcome by subgroups, Mimika district, 2014–2020.

Characteristic, Number (N) with known data	Sub-group	Treatment success*** N, %	Unfavorable outcome**** N, %	Unadjusted Odds ratio (95% CI)	P value*	Adjusted odds ratio^#^	P value
Age	0–14 years	1705	477	0.72 (0.65–0.79)	0.000	0.63 (0.55–0.71)	0.000
N = 10,087		78.1%	21.9%				
	> = 15 years	5473	2432				
		69.2%	30.8%				
Ethnicity	Papuan	5001	2007	0.96 (0.89–1.03)	0.3	0.96 (0.87–1.05)	0.384
N = 9,870		71.4%	28.7%				
	Non-Papuan	2014	848				
		70.4%	29.6%				
Residential location	Urban	5952	2277	1.16 (1.04–1.30)	0.007	1.09 (0.97–1.22)	1.114
N = 10,010		72.3	27.7%				
	Rural	1214	567				
		68.2%	31.8%				
Health facility type	Hospital	4,114	2018	0.76 (0.70–0.82)	0.000	0.54 (0.49–0.60)	0.000
N = 10,087		67.1%	32.9%				
	Primary Health Care	3,064	891				
		77.5%	22.5%				
TB type	Pulmonary	5518	2187	1.15 (1.05–1.26)	0.003	0.94 (0.84–1.05)	0.290
N = 10,087		71.6%	28.4%				
	Extra Pulmonary	1660	722				
		69.7%	30.3%				
Mode of diagnosis	Clinical diagnosis	4657	1809	0.90 (0.83–0.97)	0.008	0.83 (0.75–0.93)	0.002
N = 10,087		72.0%	28.0%				
	Bacteriologically confirmed**	2521	1100				
		69.6%	30.4%				
Record of prior TB	No past TB	6619	2625	0.76 (0.66–0.86)	0.001	0.75 (0.65–0.89)	0.000
N = 10,087		71.6%	28.4%				
	Past TB	559	284				
		66.3%	33.7%				
HIV status	HIV negative	4666	1605	0.89 (0.86–0.91)	0.000	0.91 (0.88–0.94)	0.000
N = 7,085		74.4%	25.6%				
	HIV positive	529	285				
		65.0%	35.0%				
Diabetes	Diabetes absent	3192	1064	1.10 (1.07–1.15)	0.000	0.90 (0.86–0.94)	0.000
N = 4,519		75.0%	25.0%				
	Diabetes present	203	60				
		77.2%	22.8%				

*calculated using logistic regression;

**Smear microscopy and/or Xpert positive;

***Cured and treatment completed;

****Including death, treatment failed, loss to follow up, transfer, and not recorded;

^#^ adjusted to age

## Discussion

This study provides important insights into the epidemiology of TB in Mimika district, Papua province, revealing major improvements in TB case detection over an eight-year period. The 67% increase in TB case notifications exceeded population growth of approximately 35% over the same period [9]. Substantial downturns in TB detection occurred during the COVID-19 pandemic, but the TB program showed greater resilience than reported nationally: in 2020 compared with 2019, case notifications in Mimika district decreased by 18.7% which compared to a 30.9% decline in TB case notifications in Indonesia nationally [19]. Gains made in TB program quality indicators prior to the pandemic, such as proportion of patients accessing bacteriological TB diagnostics and HIV testing, as well as the proportion achieving treatment success, were largely maintained during the first two years of the pandemic. Health system strengthening activities underway in Mimika district may have contributed to these positive performance indicators. ‘TB CEPAT’, an active case finding initiative that conducted door-to-door symptom screening in 2016–2017, is likely to have contributed to increased TB notifications whilst our CQI projects (‘STRATUM’ and ‘PRIME-TB’) have provided a package of health system strengthening activities, with a strong focus on regularly engaging, training and motivating TB program staff. The Mimika district TB program has been recognised nationally for its successes, being awarded the Best TB Program in Papua Province in 2018 and 2019 and celebrated at a national World TB Day celebration in 2019 and 2021 [20–22].

Damaging impacts of the COVID-19 pandemic on TB program performance have been reported nationally and internationally [1, 23]. Indonesian national TB case notifications dropped to 393,323 in 2020, a 31% reduction compared to 2019 and less than half the estimated caseload. While case detection rates in Mimika dropped during the first year of the pandemic, the Mimika TB program successfully returned to close to its performance before the pandemic. The case detection rate dropped from 94% in 2019 to 76% in 2020, but recovered quickly and reached above 90% in 2021. During the same time, the case detection rate at province level only increased by 2.3%, and by 6.1% at national level [17], highlighting the stronger recovery in Mimika compared to other parts of Indonesia. The findings we report from Mimika suggest that investment in program strengthening activities, including health care provider education and CQI may mitigate external impacts on the TB program. Detailed evaluation of these interventions is underway. However, further improvements are needed with regards to screening for case detection. In Mimika district, reallocation of diagnostic equipment (GeneXpert machine) and human resources to COVID-19 management, including to support a National Sports Event week in October 2021, had demonstrable negative consequences for TB screening (Fig 2). This illustrates the importance of developing strategies to build surge capacity to protect routine service delivery while responding to health emergencies.

**Fig 5:**
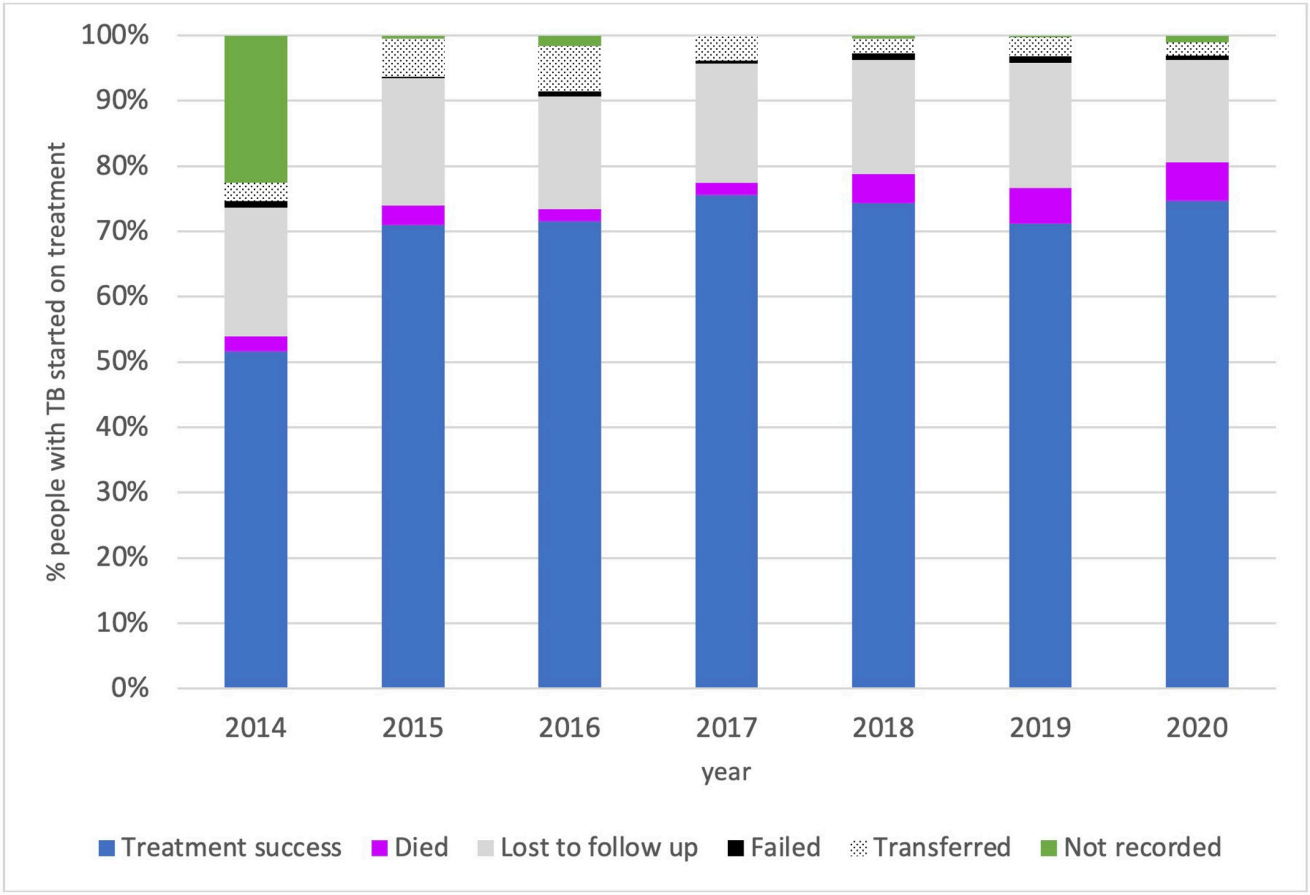
Treatment outcome among TB patients commenced on treatment, Mimika district, 2014–2020.

Our findings highlight that treatment outcomes and bacteriological confirmation are key areas for further improvement. An overall treatment success of 71.2% does not meet the target of 85% set by the Indonesian NTP or WHO [1, 24]. Furthermore, the treatment success rate did not improve from 2015 to 2020. The number of people with TB who are reported as lost to follow-up is high, especially among those receiving treatment through a hospital. Hospitals are often far from patient’s homes and have no outreach services, compared with primary care facilities which are better equipped to provide follow-up care in the community. In recognition of the need to decentralise care for treatment support, we worked with local healthcare providers to encourage treatment in primary care by implementing a referral process from hospitals to clinics. While the numbers of cases detected and treated at PHC level increased (Fig 1), the proportion of those lost to follow-up remained relatively unchanged until 2019 (Fig 5 and Table 2). A higher caseload provides additional strain on the health services to provide treatment support. Digital innovation to support active case finding, patient education, referrals, data recording and reporting is an urgent priority in settings where human resources are limited [25]. Specific challenges in Mimika district include a large and highly mobile mine worker population, and high language diversity among different ethnic groups. A large proportion of the population resident in Mimika are from other provinces in Indonesia and the high population mobility may also contribute to loss to follow-up and transfer out. More work is needed to address retention in care, such as through ensuring culturally appropriate approaches and outreach services. It is also noted that the proportion of deaths increased in 2018 and 2019. Specific reasons for this are not known, but notably, there were increases in MDR TB cases and in the proportion of TB cases being diagnosed in people aged 45–64 years. Deaths among people with TB increased further in 2020, mirroring WHO reporting on global TB-related deaths increasing by 7% during the pandemic [1]. Finally, poorer TB treatment outcomes were noted for people living with HIV including higher death rates but ART status of those with TB/HIV was not recorded highlighting the need for improved integrated care for those with comorbidities.

Bacteriological confirmation of TB was low at 35.8% compared with the national average in 2020 of 41% [17]. The only sample type reported in our study is sputum. No EPTB cases had bacteriological confirmation and hence any drug-resistant EPTB cases would have received incorrect treatment. Xpert is now increasingly used in preference to smear microscopy as per national guidelines and the use of Xpert increased during the study period. However, Xpert cartridges remain under-utilised while the analyser is required for COVID-19 testing and there was a lack of presumptive case finding. Only around 5000 of the annual allocation of more than 7000 cartridges distributed by the NTP to Mimika are being used annually. Xpert positivity among presumptive TB cases is high. Of all people tested with Xpert in 2020–2021, one in five tested positive which may suggest that not enough testing is being done (S3 Table) [26]. Programmatic planning to increase Xpert utilisation for TB testing is of particular importance while the competing need for COVID-19 testing with Xpert persists. Rifampicin resistance was 7.3% among those tested with Xpert in 2021, a concerning problem for Mimika given the high reliance on clinical diagnoses and empirical therapy with a standard drug-susceptible TB regimen. Xpert for EPTB diagnosis was unavailable in Mimika during this study due to lack of equipment (centrifuge and tubes), training or biosafety cabinet access for level 3 sample processing, and lack of finance from the NTP or health insurance to cover these costs.

Xpert clearly has high appeal given higher yield and lesser workload than microscopy and is now recommended in Indonesia for broad use. However, as utilisation of smear microscopy decreases and Xpert increases, there is a need to maintain microscopy expertise since this is still recommended for monitoring treatment response. By 2021, 27.7% of presumptive TB cases were diagnosed on microscopy and 63.5% using Xpert.

TB case finding in children is a marker of the quality of TB program performance. The proportion of TB cases in Mimika aged less than 15 years is consistently higher than national data. In 2020, 21.3% of TB cases in Mimika district were children aged 0–14 years, compared to only 9% nationally [2]. Infant BCG vaccination coverage in Mimika is low only 60.7% in 2020 and 64.5% in 2021. The high proportion of cases reported among children is similar to that reported from neighbouring Papua New Guinea (25% of cases aged less than 15 years), where TB is also highly endemic and where children make up a large proportion of the population [27]. Census data indicate that children comprise approximately 35% of the population in Mimika district compared to 23% in Indonesia overall. Only 5.6% of children were confirmed bacteriologically in this cohort highlighting the need for better TB diagnostics for children, especially young children with paucibacillary disease. Most (58%) of the bacteriologically confirmed TB in was diagnosed by Xpert and the proportion of bacteriologically confirmed child TB has increased since the introduction of Xpert, but remains low. Sputum induction or gastric aspirates for testing with Xpert can be done at the district hospital but these procedures require hospitalisation and are not routine for children with mild TB symptoms in the outpatient clinic. Furthermore, the WHO has recommended avoiding sputum induction to prevent airborne transmission during the COVID-19 pandemic. Recently updated WHO child TB guidelines (2022) recommend alternative samples for Xpert testing such as nasopharyngeal aspirates or stool [28]. The diagnosis of TB in children is usually clinical. The Indonesian pediatric TB scoring system is widely used and 52.1% of child TB patients in Mimika had a score ≥6, which indicates the need for TB treatment [17]. However, the scoring system includes tuberculin skin test results (largely unavailable at our study site) and chest X-ray also (limited in availability, only at hospitals requiring a referral from primary care) which limit its diagnostic value.

The main limitation of this study is incomplete data capture especially in the first year; however, we undertook stringent cross-checking to minimise missing data and validate entries, applied consistently across all years of the study. Ethnicity is not captured in the TB electronic records; some errors in assigning ethnicity could have occurred. We did not have access to directly comparable national TB datasets and therefore comparisons between program performance in Mimika district with other parts of Indonesia were only able to be descriptive. Compared to the other 28 districts in Papua, Mimika district ranked first in TB case finding in 2020 and 2021 [17]. While the population comprised 7.3% of the total population of Papua, it is estimated that about 10% of TB cases will be notified from Mimika district. However, in 2021, Mimika district was able to contribute to 18.4% of total TB case finding in Papua [17]. Strengths of this study include the highly comprehensive dataset from an under-reported, high burden setting, uniquely able to track program performance over a long period incorporating the unexpected disruption caused by COVID-19.

In summary, the implementation of several dedicated TB case finding and health system strengthening activities substantially improved TB case detection in Mimika District. Treatment success rate was sustained despite the increased burden that TB case numbers posed to the health services, but improvements in treatment outcome are needed. The analysis shows the negative impact of the COVID-19 pandemic on TB surveillance but there are already encouraging signs of recovery in case detection suggesting resilience in the TB services. There are clear areas for ongoing investment, including greater availability and uptake of rapid and accurate diagnostics, which international collaborative work in this study setting continues to address.

## Supporting information

S1 Table Laboratory testing among people diagnosed with TB, Mimika district, 2014–2021. (DOCX)

S2 Table TB treatment outcome in subgroups by year. (DOCX)

S3 Table Investigation for possible tuberculosis in symptomatic children and adults, 2019–2021. (DOCX)

S4 Table Child TB diagnosis algorithm. (DOCX)

S1 Fig TB treatment outcome in subgroups. (TIF)

S2 Fig Mortality (death from any cause during TB treatment) by age group over time. (TIF)

S3 Fig Overall mortality by subgroups. (TIF)
