# Supplementary material for: Impacts of tuberculosis services strengthening and the COVID-19 pandemic on case detection and treatment outcomes in Mimika District, Papua, Indonesia: 2014–2021
Source: PLOS Glob Public Health. 2022 Sep 30;2(9):e0001114. doi: 10.1371/journal.pgph.0001114 (PMC10021881; doi:10.1371/journal.pgph.0001114)
Supplement: S1 Table — (DOCX) [file pgph.0001114.s001.docx]

**S1 Table: Laboratory testing among people diagnosed with TB, Mimika district, 2014-2021**

| **Year** | **2014** | **2015** | **2016** | | **2017** | **2018** | | **2019** | | **2020** | | **2021** | | **Total** | |
| --- | --- | --- | --- | --- | --- | --- | --- | --- | --- | --- | --- | --- | --- | --- | --- |
| **TB caseload** | **920** | **1078** | **1506** | | **1567** | **1759** | | **1796** | | **1461** | | **1716** | | **11803** | |
| **Smear microscopy** | | | | | | | | | | | | | | | |
| Numbers examined  (% of caseload) | 705  76.6 | 778  72.2 | 1096  72.8 | | 1155  73.7 | 1176  66.9 | | 866  48.2 | | 598  40.9 | | 440  25.6 | | 6814  577 | |
| Smear positive  (% of caseload) | 353  38.4% | 361  33.5% | 486  32.3 | | 542  34.6 | 526  29.9 | | 304  16.9 | | 222  15.2 | | 141  8.2 | | 2935  24.9 | |
| Smear positivity rate  % positive/examined | 50.1% | 46.4 | 44.3 | | 46.9 | 44.7 | | 35.1 | | 37.1 | | 32.0 | | 43.1 | |
| Children examined  (% of child TB cases) | 10  5.9 | 8  4.2 | 14  4.2 | | 32  8.9 | 62  14.7 | | 38  9.6 | | 34  10.9 | | 13  3.2 | | 211  8.2 | |
| Smear positive children  (% of examined) | 5  50.0 | 3  37.5 | 4  28.5 | | 9  28.1 | 14  22.6 | | 11  28.9 | | 7  20.5 | | 3  23.1 | | 56  26.5 | |
| Adults examined  (% of adult TB cases) | 695  92.8 | 770  86.8 | 1082  92.2 | | 1123  93.0 | 1114  83.3 | | 828  59.1 | | 564  49.0 | | 427  32.5 | | 6603  71.6 | |
| Smear positive adults  (% of examined) | 348  50.1 | 358  46.5 | 482  44.5 | | 533  47.5 | 512  45.9 | | 293  35.4 | | 215  38.1 | | 138  32.3 | | 2879  43.6 | |
| Adult PTB examined  (% of adult PTB cases) | 576  95.8 | 646  98.8 | 910  99.9 | | 969  99.4 | 1005  93.4 | | 507  53.3 | | 532  53.0 | | 408  34.6 | | 5553  75.5 | |
| Smear positive adult PTB cases (% positive) | 339  58.8 | 353  54.6 | 475  52.2 | | 517  53.4 | 501  49.9 | | 291  57.4 | | 215  40.4 | | 138  33.8 | | 2829  50.9 | |
| **Xpert MTB/RIF** | | | | | | | | | | | | | | | |
| Numbers examined  (% of caseload) | Not recorded | | | 29  1.9 | | | 337  19.2 | | 677  37.7 | | 563  38.5 | | 908  52.9 | | 2519  21.3 |
| Xpert positive  (% of caseload) |  |  |  | 16  1.0 | | | 203  11.5 | | 472  26.3 | | 380  26.0 | | 517  30.1 | | 1587  13.4 |
| Xpert positivity rate  % positive/examined |  |  |  | 55.1 | | | 60.2 | | 69.8 | | 67.5 | | 56.9 | | 63.0 |
| Children examined  (% of child TB cases) |  |  |  | 3  0.8 | | | 42  10.0 | | 38  9.6 | | 49  15.8 | | 61  15.1 | | 193  7.5 |
| Xpert positive children  (% of examined) |  |  |  | 1*  33.3 | | | 10  23.8 | | 14  36.8 | | 15  30.6 | | 22  36.1 | | 62  32.1 |
| **Bacteriologically confirmed** | | | | | | | | | | | | | | | |
| Bacteriology confirmed TB cases (% of caseload) | 353 | 361 | 486 | | 545 | 620 | | 682 | | 574 | | 609 | | 4230 | |
|  | 38.4 | 33.5 | 32.3 | | 34.8 | 35.3 | | 38.0 | | 39.3 | | 35.5 | | 35.8 | |
| Clinically diagnosed TB cases (% of caseload) | 567 | 717 | 1020 | | 1022 | 1139 | | 1114 | | 887 | | 1107 | | 7573 | |
|  | 61.6 | 66.5 | 67.7 | | 65.2 | 64.7 | | 62.0 | | 60.7 | | 64.5 | | 64.2 | |
| Bacteriology confirmed adult PTB (% of PTB) | 339 | 353 | 475 | | 519 | 580 | | 643 | | 552 | | 585 | | 4046 | |
|  | 56.4 | 54.0 | 52.1 | | 53.2 | 53.9 | | 67.6 | | 55.0 | | 49.6 | | 55.0 | |
| Bacteriology confirmed child PTB (% of PTB) | 5 | 3 | 3 | | 8 | 20 | | 22 | | 21 | | 24 | | 106 | |
|  | 4.1 | 2.2 | 1.5 | | 3.2 | 6.6 | | 7.8 | | 8.6 | | 6.9 | | 5.6 | |
| **Rifampicin resistance*** | | | | | | | | | | | | | | | |
| Rifampicin-resistant TB cases (% of caseload) | 0  0 | 3  0.3 | 2  0.1 | | 6  0.4 | 13  0.7 | | 36  2.0 | | 21  1.4 | | 26  3.1 | | 107  0.9 | |
| % Rifampicin resistant of all Xpert tests | 0 | 100 | 100 | | 20.7 | 3.9 | | 5.3 | | 3.7 | | 7.3 | | 4.2 | |
| % Rifampicin resistant of MTB+ Xpert tests | 0 | 100 | 100 | | 37.5 | 6.3 | | 7.6 | | 5.5 | | 5.0 | | 6.7 | |

* prior to availability of GeneXpert in Mimika in 2018, selected specimens from people with presumptive MDR TB were sent to the provincial reference laboratory in the capital, Jayapura.
