# Supplementary material for: Impacts of tuberculosis services strengthening and the COVID-19 pandemic on case detection and treatment outcomes in Mimika District, Papua, Indonesia: 2014–2021
Source: PLOS Glob Public Health. 2022 Sep 30;2(9):e0001114. doi: 10.1371/journal.pgph.0001114 (PMC10021881; doi:10.1371/journal.pgph.0001114)
Supplement: S2 Table — (DOCX) [file pgph.0001114.s002.docx]

**S2 Table: TB treatment outcome in subgroups by year**

| **Year** | **2014** | **2015** | **2016** | **2017** | **2018** | **2019** | **2020** | **Total** |
| --- | --- | --- | --- | --- | --- | --- | --- | --- |
| **Total cases treated** | **920** | **1078** | **1506** | **1567** | **1759** | **1796** | **1461** | **11803** |
| **Treatment success among sub-groups** | **Numbers with success/Total number in sub-group**  **%** | | | | | | | |
| Children (0-14 years) | 116/171 | 159/191 | 264/332 | 288/360 | 341/421 | 307/309 | 230/311 | 1705/2182 |
|  | 67.8 | 83.3 | 79.5 | 80.0 | 81.0 | 77.5 | 74.0 | 78.1 |
| Papuan ethnicity | 358/632 | 557/799 | 748/1037 | 784/1043 | 908/1227 | 894/1266 | 752/1004 | 5001/7008 |
|  | 56.7 | 69.7 | 72.1 | 75.2 | 74.0 | 70.6 | 74.9 | 71.4 |
| Bacteriology confirmed | 168/353 | 262/361 | 348/486 | 419/545 | 438/620 | 469/682 | 417/574 | 2521/3621 |
|  | 47.6 | 72.6 | 71.6 | 76.9 | 70.7 | 68.8 | 72.7 | 69.6 |
| EPTB | 130/197 | 196/286 | 284/399 | 256/341 | 264/381 | 380/564 | 150/214 | 1660/2382 |
|  | 66.0 | 68.5 | 71.2 | 75.1 | 69.3 | 67.4 | 70.1 | 69.7 |
| HIV positive | 37/74 | 99/146 | 96/166 | 89/124 | 92/125 | 45/78 | 71/101 | 529/814 |
|  | 50.0 | 67.8 | 57.8 | 71.8 | 73.6 | 57.7 | 70.3 | 65.0 |
| Diabetes | Not recorded | | | 33/43 | 56/75 | 59/71 | 47/61 | 203/263 |
|  |  |  |  | 76.7 | 74.7 | 83.1 | 77.1 | 77.2 |
| History of TB treatment | 23/44 | 32/51 | 59/87 | 110/148 | 119/185 | 108/177 | 108/151 | 559/843 |
|  | 52.3 | 62.8 | 67.8 | 74.3 | 64.3 | 61.0 | 71.5 | 66.3 |
| Rural residence | 61/137 | 137/202 | 210/290 | 205/276 | 197/283 | 230/339 | 174/254 | 1214/1781 |
|  | 44.5 | 67.8 | 72.4 | 74.3 | 69.6 | 67.9 | 68.5 | 68.2 |
| Urban residence | 414/735 | 626/869 | 865/1213 | 978/1291 | 1109/1467 | 1049/1455 | 911/1199 | 5952/8229 |
|  | 56.3 | 72.0 | 71.3 | 75.8 | 75.6 | 72.1 | 76.0 | 72.3 |
| Hospital | 317/617 | 530/801 | 702/1083 | 713/983 | 706/997 | 642/974 | 504/722 | 4114/6132 |
|  | 51.4 | 66.2 | 67.6 | 72.5 | 70.8 | 65.9 | 69.8 | 67.1 |
| Primary Health Center | 158/303  52.1 | 236/277  85.2 | 376/468  80.3 | 470/584  80.5 | 600/762  78.7 | 638/822  77.6 | 586/739  79.3 | 3064/3955  77.5 |
| **Deaths among sub-groups** | **Numbers of deaths/Total number in sub-group with known outcome**  **%** | | | | | | | |
| Children  (0-14 years) | 1/171 | 2/191 | 1/332 | 2/360 | 6/421 | 6/396 | 13/311 | 31/2182 |
|  | 0.6 | 1.1 | 0.3 | 0.6 | 1.4 | 1.5 | 4.2 | 1.4 |
| Adults  (15-64 years) | 19/735 | 28/863 | 27/1137 | 28/1174 | 69/1307 | 87/1364 | 68/1121 | 326/7701 |
|  | 2.6 | 3.2 | 2.4 | 2.4 | 5.2 | 6.4 | 6.1 | 4.2 |
| Elderly  (>64 years) | 1/14 | 2/24 | 0 | 2/33 | 4/31 | 4/36 | 7/29 | 20/204 |
|  | 7.1 | 8.3 | 0 | 6.1 | 12.9 | 11.1 | 24.1 | 9.8 |
| Bacteriologically confirmed | 8/353 | 10/361 | 10/486 | 17/545 | 32/620 | 54/682 | 34/574 | 165/3621 |
|  | 2.3 | 2.8 | 2.1 | 3.1 | 5.2 | 7.9 | 5.9 | 4.6 |
| Papuan | 15/632 | 23/799 | 14/1037 | 20/1043 | 50/1227 | 62/1266 | 52/1004 | 236/7008 |
|  | 2.4 | 2.9 | 1.3 | 1.9 | 4.1 | 4.9 | 5.2 | 3.4 |
| Non-Papuan | 6/274 | 9/250 | 11/391 | 7/450 | 27/521 | 35/525 | 36/451 | 131/2862 |
|  | 2.2 | 3.6 | 2.8 | 1.6 | 5.2 | 6.7 | 8.0 | 4.6 |
| EPTB | 4/197 | 10/286 | 5/399 | 6/341 | 23/381 | 28/564 | 18/214 | 94/2.382 |
|  | 2.0 | 3.5 | 1.3 | 1.7 | 6.0 | 5.0 | 8.4 | 4.0 |
| HIV positive | 1/74 | 5/146 | 2/166 | 4/124 | 2/125 | 12/78 | 10/101 | 36/814 |
|  | 1.3 | 3.4 | 1.2 | 3.2 | 1.6 | 15.4 | 9.9 | 4.4 |
| Diabetes | Not recorded | | | 2/43 | 7/75 | 3/71 | 4/61 | 16/263 |
|  |  |  |  | 4.7 | 9.3 | 4.2 | 6.7 | 6.1 |
| Past TB treatment | 5/44 | 3/51 | 2/87 | 5/148 | 15/185 | 21/177 | 13/151 | 64/843 |
|  | 11.4 | 5.9 | 2.3 | 3.4 | 8.1 | 11.9 | 8.6 | 7.6 |
