# Supplementary material for: Impacts of tuberculosis services strengthening and the COVID-19 pandemic on case detection and treatment outcomes in Mimika District, Papua, Indonesia: 2014–2021
Source: PLOS Glob Public Health. 2022 Sep 30;2(9):e0001114. doi: 10.1371/journal.pgph.0001114 (PMC10021881; doi:10.1371/journal.pgph.0001114)
Supplement: S3 Table — (DOCX) [file pgph.0001114.s003.docx]

**S3 Table: Investigation for possible tuberculosis in symptomatic children and adults, 2019-2021**

| **Year** | **2019** | **2020** | **2021** | **Total** |
| --- | --- | --- | --- | --- |
| **Total presumptive TB cases** | **7745** | **4808** | **4899** | **17452** |
| Laboratory test performed: smear or GeneXpert  % of total presumptive TB | 6562  84.7 | 4333  90.1 | 4225  86.2 | 15120  86.6 |
| Smear microscopy performed  % of total presumptive TB | 3960  51.1 | 2231  46.4 | 1358  27.7 | 7549  43.3 |
| Positive smear microscopy  % of smear microscopy performed | 263  6.6 | 226  10.1 | 158  11.6 | 647  3.7 |
| GeneXpert MTB/RIF performed  % of total presumptive TB | 3579  46.2 | 2268  47.2 | 3112  63.5 | 8959  51.3 |
| Positive GeneXpert MTB/RIF  % of GeneXpert performed | 528  14.8 | 442  19.5 | 639  20.5 | 1609  18.0 |
| Rifampicin resistant cases detected  % of GeneXpert positive | 36  6.8 | 21  4.8 | 26  4.1 | 83  5.2 |
|  | | | | |
| **Presumptive pulmonary TB in adults** | **6594** | **3703** | **3348** | **13645** |
| Laboratory diagnostic test performed  % of presumptive adult PTB | 6068  92.0 | 3614  97.6 | 3255  97.2 | 12937  94.8 |
| Smear microscopy performed  % of presumptive adult PTB | 3763  57.1 | 1989  53.7 | 1032  30.8 | 6784  497 |
| Positive smear microscopy  % of smear microscopy performed | 253  6.7 | 217  10.9 | 141  13.7 | 611  4.5 |
| GeneXpert test performed  % of presumptive adult PTB | 3247  49.2 | 1783  48.2 | 2430  72.6 | 7460  54.7 |
| GeneXpert positive  % of GeneXpert performed | 511  15.7 | 400  22.4 | 556  22.9 | 1467  19.7 |
|  |  |  |  |  |
| **Presumptive TB in all children (0-14 years)** | **955** | **584** | **623** | **2162** |
| Presumptive pulmonary TB in children  % of all presumptive TB in children | 849 | 455 | 489 | 1793 |
|  | | | | |
| **0-4 years** | **392** | **265** | **298** | **955** |
| Laboratory diagnostic test performed  % of young children with presumptive TB | 80  20.4 | 117  44.1 | 76  25.5 | 273  28.6 |
| Smear microscopy performed  % of young children with presumptive TB | 12  3.1 | 10  3.8 | 5  1.7 | 27  2.8 |
| Positive smear microscopy  % of smear microscopy performed | 0  0 | 1  10 | 1  20 | 2  0.2 |
| GeneXpert test performed  % of young children with presumptive TB | 70  17.9 | 108  40.8 | 71  23.8 | 249  26.1 |
| GeneXpert positive  % of GeneXpert tested | 3  4.3 | 6  5.6 | 3  4.2 | 12  4.8 |
| **5-14 years** | **563** | **319** | **325** | **1207** |
| Laboratory diagnostic test performed  % of young children with presumptive TB | 316  56.1 | 190  59.6 | 196  60.3 | 702  58.2 |
| Smear microscopy performed  % of young children with presumptive TB | 129  22.9 | 63  19.8 | 29  8.9 | 221  18.3 |
| Positive smear microscopy  % of smear microscopy performed | 10  7.8 | 6  9.5 | 2  6.9 | 18  1.5 |
| GeneXpert test performed  % of young children with presumptive TB | 205  36.4 | 129  40.4 | 170  52.3 | 504  41.8 |
| GeneXpert positive  % of GeneXpert tested | 13  6.3 | 14  10.9 | 20  11.8 | 47  9.3 |
| **Rifampicin resistance** | | | | |
| GeneXpert Rifampicin resistance detected  % of GeneXpert positive overall | 36  6.8 | 21  4.8 | 26  4.1 | 83  5.2 |
| GeneXpert Rifampicin resistance detected in children (0-14 years)  % of GeneXpert positive in children | 0  0 | 1  5% | 1  4.3% | 2  3.4% |
