# Supplementary material for: Impacts of tuberculosis services strengthening and the COVID-19 pandemic on case detection and treatment outcomes in Mimika District, Papua, Indonesia: 2014–2021
Source: PLOS Glob Public Health. 2022 Sep 30;2(9):e0001114. doi: 10.1371/journal.pgph.0001114 (PMC10021881; doi:10.1371/journal.pgph.0001114)
Supplement: S4 Table — (DOCX) [file pgph.0001114.s004.docx]

**S4 Table: Child TB scoring system**

| **Parameter** | **0** | **1** | **2** | **3** | **Skor** |
| --- | --- | --- | --- | --- | --- |
| Contact with active TB patient | Not clear |  | Contact with a smear negative TB patient. | Contact with smear positive TB patient |  |
| Tuberkulin skin test | Negatif |  |  | Positive |  |
| Nutrition status |  | Weight/height<90%  or  Weight/age<80% | Malnutrition or  Weight/heigh<70% or weight/age<60% |  |  |
| Fever of unknown origin |  | >2 weeks |  |  |  |
| Chronic cough |  | >3 weeks |  |  |  |
| Lymph enlargement |  | >1, >1 lymph, and no pressure pain |  |  |  |
| Bone and joint |  | Enlargement |  |  |  |
| Chest x-ray | Normal | Suggestive TB |  |  |  |
| Maximal total score: | | | | |  |
