# Supplementary figures and images for: Impacts of tuberculosis services strengthening and the COVID-19 pandemic on case detection and treatment outcomes in Mimika District, Papua, Indonesia: 2014–2021
Source: PLOS Glob Public Health. 2022 Sep 30;2(9):e0001114. doi: 10.1371/journal.pgph.0001114 (PMC10021881; doi:10.1371/journal.pgph.0001114)

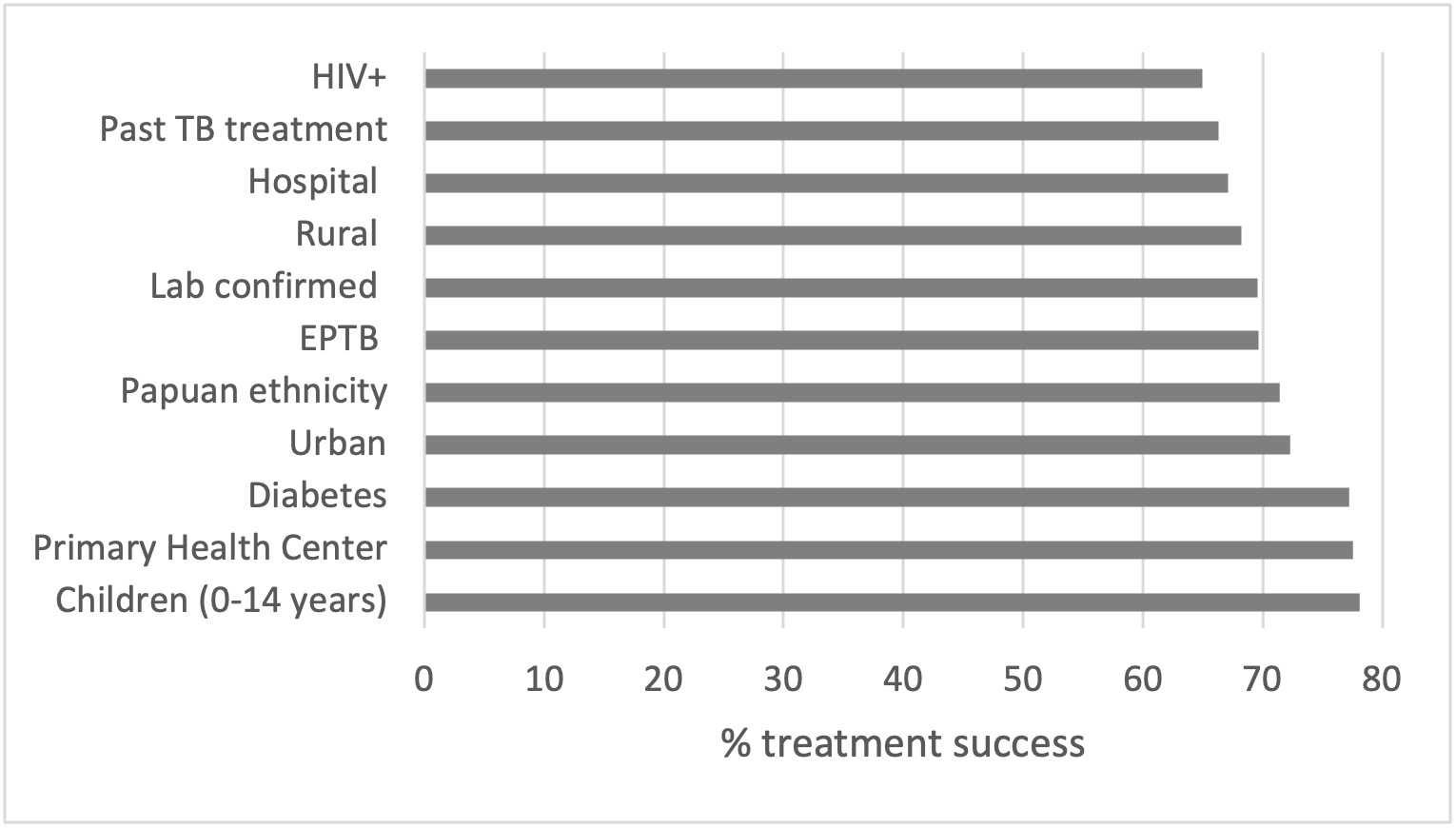

Supplement: S1 Fig — (TIF) [file pgph.0001114.s005.tif]

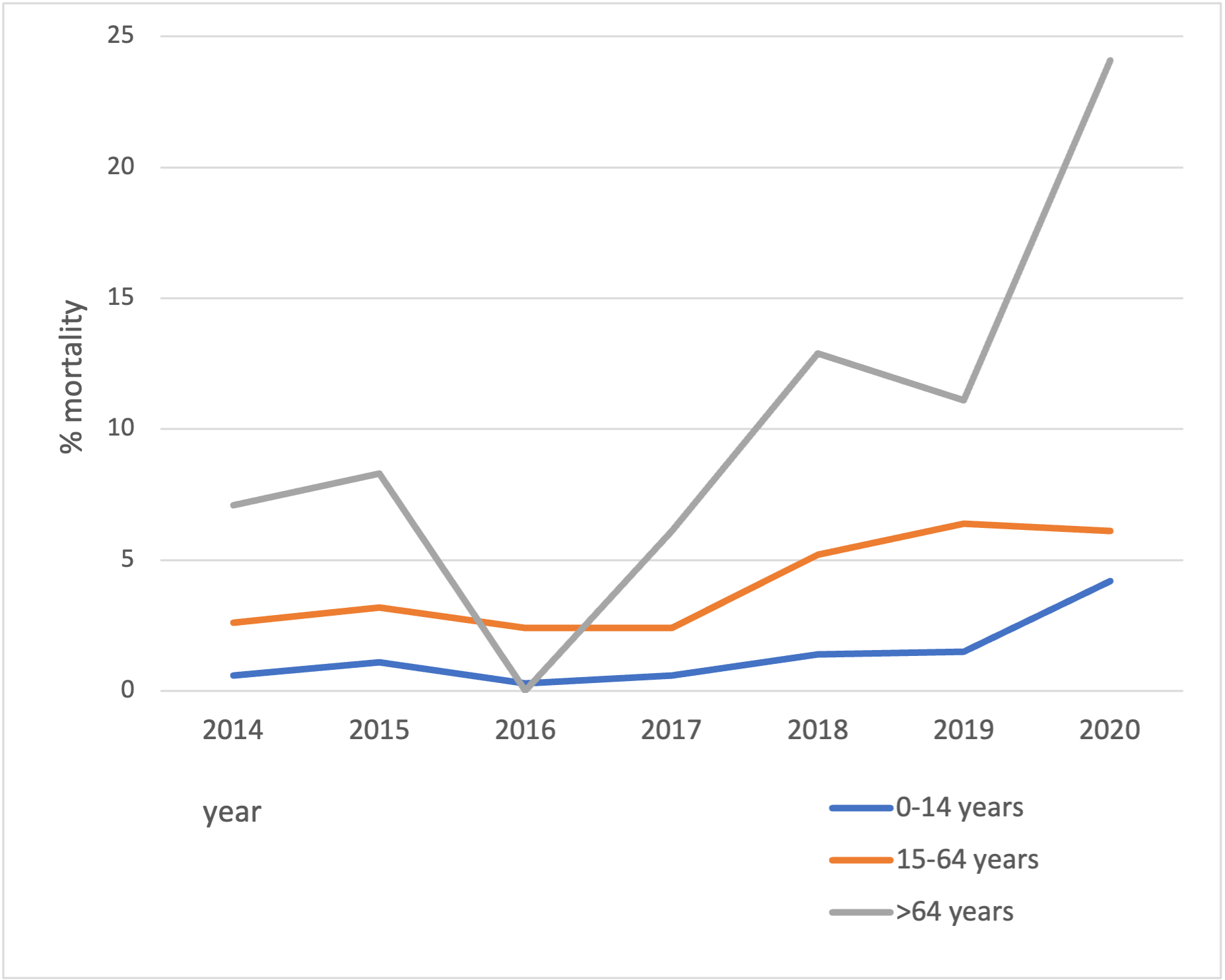

Supplement: S2 Fig — (TIF) [file pgph.0001114.s006.tif]

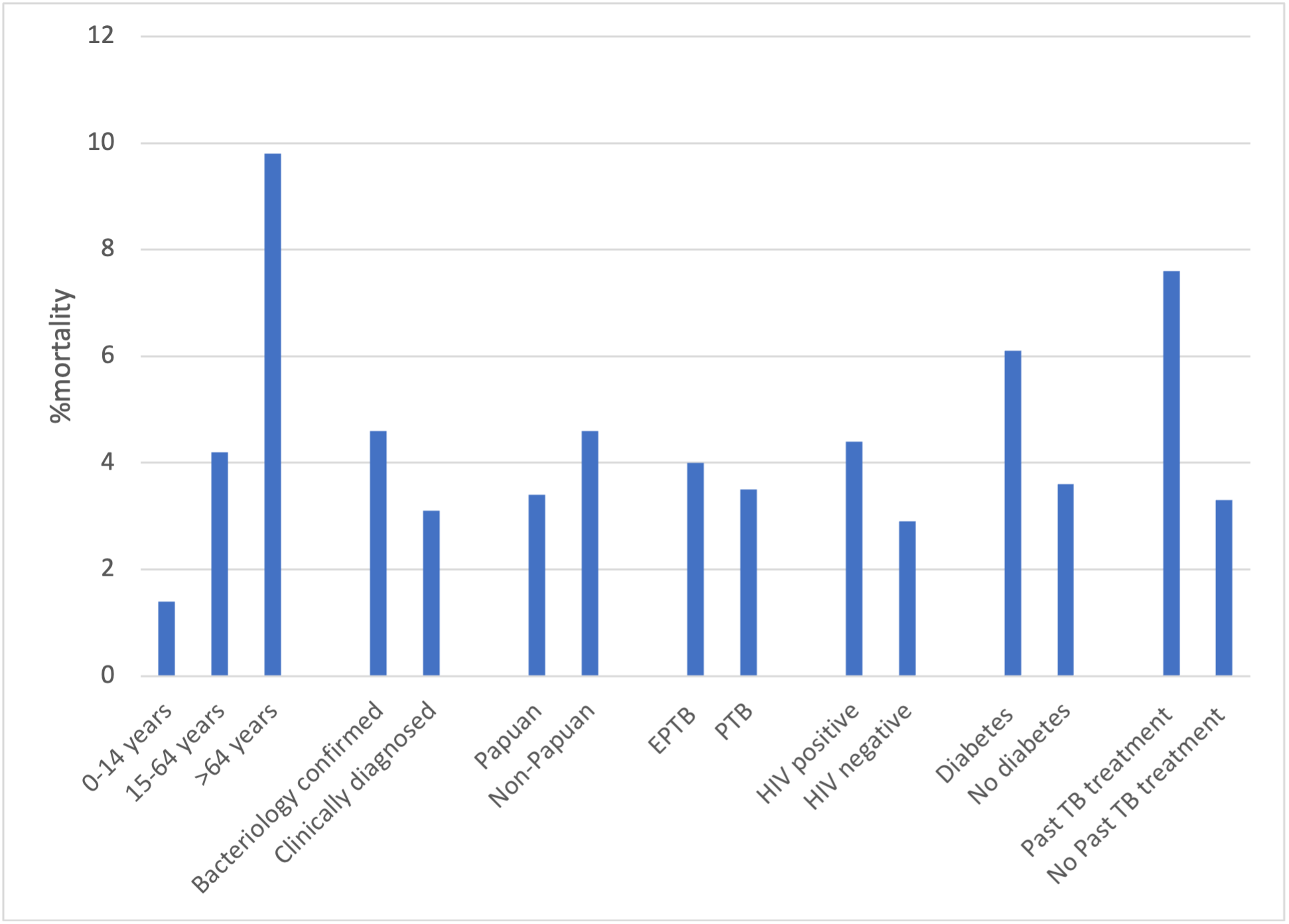

Supplement: S3 Fig — (TIF) [file pgph.0001114.s007.tif]
